# Ultrahigh-Q Topological Mirror Heterostructure for High-Resolution Thermal Sensing in One-Dimensional Photonic Crystals

**DOI:** 10.3390/nano16211399

**Published:** 2026-10-31

**Authors:** Mohamed Saleh M. Esmail, Yassine Bouazzi, Mabrouk Touahmia, Zied Elleuch, Sayed El. Soliman

**Affiliations:** 1 Basic Science Department, Faculty of Engineering, Misr University for Science and Technology, Giza 12588, Egypt; mohamed.esmail@must.edu.eg; 2 Department of Industrial Engineering, College of Engineering, University of Ha’il, Ha’il 81481, Saudi Arabia; yassine.bouazzi@enit.utm.tn; 3 Photovoltaic and Semiconductor Materials Laboratory, National Engineering School of Tunis, University of Tunis El Manar, Tunis 1002, Tunisia; 4 Department of Civil Engineering, College of Engineering, University of Ha’il, Ha’il 81481, Saudi Arabia; m.touahmia@uoh.edu.sa; 5 Computer Science Department, Applied College, University of Ha’il, Ha’il 81481, Saudi Arabia; zi.elleuch@uoh.edu.sa; 6 Faculty of Basic and Applied Sciences, Egypt-Japan University of Science and Technology, Alexandria 21934, Egypt; 7 Physics Department, Faculty of Science, Assiut University, Assiut 10587, Egypt

**Keywords:** topological mirror heterostructure, one-dimensional photonic crystals, thermal sensing, topological edge states, ultrahigh-Q resonance, figure of merit

## Abstract

High-resolution optical thermal sensors are attracting increasing interest due to their potential for compact integration, electromagnetic immunity, and enhanced spectral precision. In this work, a topological mirror heterostructure based on a one-dimensional photonic crystal (1DPC) is proposed for thermal sensing applications. The structure incorporates alternating silicon/glycerin photonic layers with a glycerin defect cavity, combining the large thermo-optic response of glycerin with the strong field localization of topological edge states in a topological mirror configuration. Finite element method (FEM) simulations reveal the formation of ultranarrow resonant modes with near-unity transmission inside the photonic bandgap. The proposed topological mirror heterostructure achieves exceptional thermal sensing performance through ultranarrow resonant linewidths and strong optical confinement, resulting in ultrahigh quality factors and figures of merit. The optimized design exhibits a thermal sensitivity of −0.1177 nm/°C, a quality factor approaching 1.8 × 10^8^, and a figure of merit of 1.53 × 10^4^ °C^−1^. The proposed platform offers a promising approach to compact photonic thermal sensors that require high spectral resolution and enhanced sensing efficiency.

## 1. Introduction

Accurate and high-resolution temperature sensing is essential in biomedical diagnostics, environmental monitoring, aerospace systems, and industrial process control [1,2,3]. Conventional thermal sensors, including thermocouples, thermistors, and resistance temperature detectors, are widely used because of their simplicity and reliability [4,5]. However, these devices often suffer from electromagnetic interference, limited miniaturization capability, and relatively slow response times in micro- and nanoscale applications [6]. Optical thermal sensors have therefore attracted increasing attention owing to their immunity to electromagnetic noise, compact integration capability, remote operation, and rapid response characteristics [7,8]. Recent advances in nanoscale optical engineering have also shown that structural design can be used to control light–matter interactions to achieve desired sensing functionalities. Surface-enhanced Raman scattering (SERS) has been used for label-free molecular discrimination with engineered two-dimensional chiral superlattices, demonstrating the broader connection between nanoscale architecture and optical sensing performance [9].

Photonic crystals (PCs) are promising platforms for optical sensing because of their ability to manipulate and localize electromagnetic waves through photonic bandgap engineering [10,11]. In particular, one-dimensional photonic crystals (1DPCs) support defect and resonant modes that are highly sensitive to temperature-induced refractive-index [12,13,14,15,16]. Introducing defect layers inside 1DPCs enables strong light localization within the photonic bandgap, thereby enhancing thermal sensing performance [12,14,17,18]. Nevertheless, conventional defect-based resonances often exhibit broader linewidths and sensitivity to fabrication imperfections and structural disorder, which can limit spectral resolution and sensing efficiency [19]. To overcome these limitations, topological photonic structures have recently emerged as attractive candidates for high-performance optical sensing [20,21,22]. In 1DPCs, localized edge states can arise at the interface between photonic lattices with distinct topological properties, such as different Zak phases [23,24]. These topological edge states (TESs) exhibit strong optical confinement and enhanced spectral localization within the photonic bandgap, enabling ultranarrow resonance linewidths and high-quality (*Q*) optical modes. Such properties are highly desirable for high-resolution thermal sensing applications that require strong spectral selectivity and stable resonant behavior.

Many studies have also highlighted the versatility of photonic crystal heterostructures for tailoring optical responses by manipulating interfaces and materials. For instance, in photonic crystal heterojunctions involving Weyl semimetals, nonreciprocal optical Tamm states were reported by Xu et al. [25], whereas He et al. reported tunable thermal emission in one-dimensional photonic crystal heterojunctions, including graphene [26]. In a recent study, unidirectional perfect absorption in a topological photonic crystal heterostructure, due to topological interface states, and the optical response of Weyl semimetals were described by Deng et al. [27]. Moreover, recent studies have demonstrated the potential of both conventional and topological 1DPC thermal sensors [16,24,28,29,30,31]. Liquid-crystal-based defect structures have achieved high thermal sensitivity due to their strong thermo-optic response [30], while topological photonic heterostructures have demonstrated ultrahigh-Q resonances and enhanced figures of merit through strong interface-state confinement [17,23]. However, achieving simultaneously strong thermo-optic tunability, ultranarrow resonance linewidths, and high spectral selectivity remains a major challenge in photonic thermal sensing.

Glycerin is particularly attractive for thermal sensing applications because of its relatively large thermo-optic coefficient, liquid-state tunability, and compatibility with infiltration-based photonic fabrication techniques [31]. Temperature-dependent variations in the refractive index of glycerin can effectively modulate the optical path length and resonance position of PC structures. However, the integration of glycerin within topological PC architectures, particularly in 1D topological mirror heterostructures, has not yet been extensively explored. Combining the strong thermo-optic response of glycerin with the enhanced optical confinement of topological edge states, therefore, offers a promising route toward high-resolution thermal sensing with ultranarrow resonance linewidths and enhanced figure of merit (FOM) values. Our previous study investigated thermal sensing via coupled topological interface states in a one-dimensional photonic crystal mirror heterostructure [23]. In that configuration, the resonant response arose predominantly from coupling and hybridization between interface-localized topological modes. In this work, a topological mirror heterostructure based on a 1DPC is proposed for high-resolution thermal sensing. The structure consists of alternating silicon and glycerin layers that support strongly localized TESs within the photonic bandgap. The thermal sensing characteristics of the proposed device are investigated using the finite element method (FEM). The present work advances this proposed design by introducing a finite glycerin-filled defect cavity between the topological mirror sections. This modification produces a defect-engineered cavity resonance in which the optical field is concentrated within the temperature-responsive liquid region, while the surrounding topological mirrors control leakage from the cavity. Thus, the present mechanism combines cavity-enhanced thermo-optic interaction with topological-mirror confinement and is physically distinct from approaches that rely solely on coupled interface states.

The proposed topological mirror heterostructure achieves exceptional thermal sensing performance through ultranarrow resonant linewidths and strong optical confinement, resulting in ultrahigh *Q* factors and FOM values. The effects of defect-layer thickness and mirror periodicity on the sensing performance are systematically analyzed. The results obtained demonstrate the potential of topological photonic structures for compact, integrated, and high-resolution thermal sensing platforms. The principal distinction of the present structure is the combination of a topological interface-state mechanism, a glycerin-filled thermal cavity, normal-incidence spectral interrogation, and good thermal sensitivity within a compact one-dimensional multilayer configuration. The calculated ultrahigh quality factor represents the predicted leakage-limited response of the lossless model. The novelty of the present study does not lie in introducing the topological mirror-heterostructure concept itself. Its contribution is the incorporation of a glycerin-filled defect cavity and the joint optimization of the glycerin-layer thickness and mirror periodicity. This approach preserves a thermal sensitivity comparable to that of our previous coupled-edge-state sensor while producing substantial expected improvements in resonance confinement and linewidth-dependent performance.

## 2. Structure and Theory

**Figure 1:**
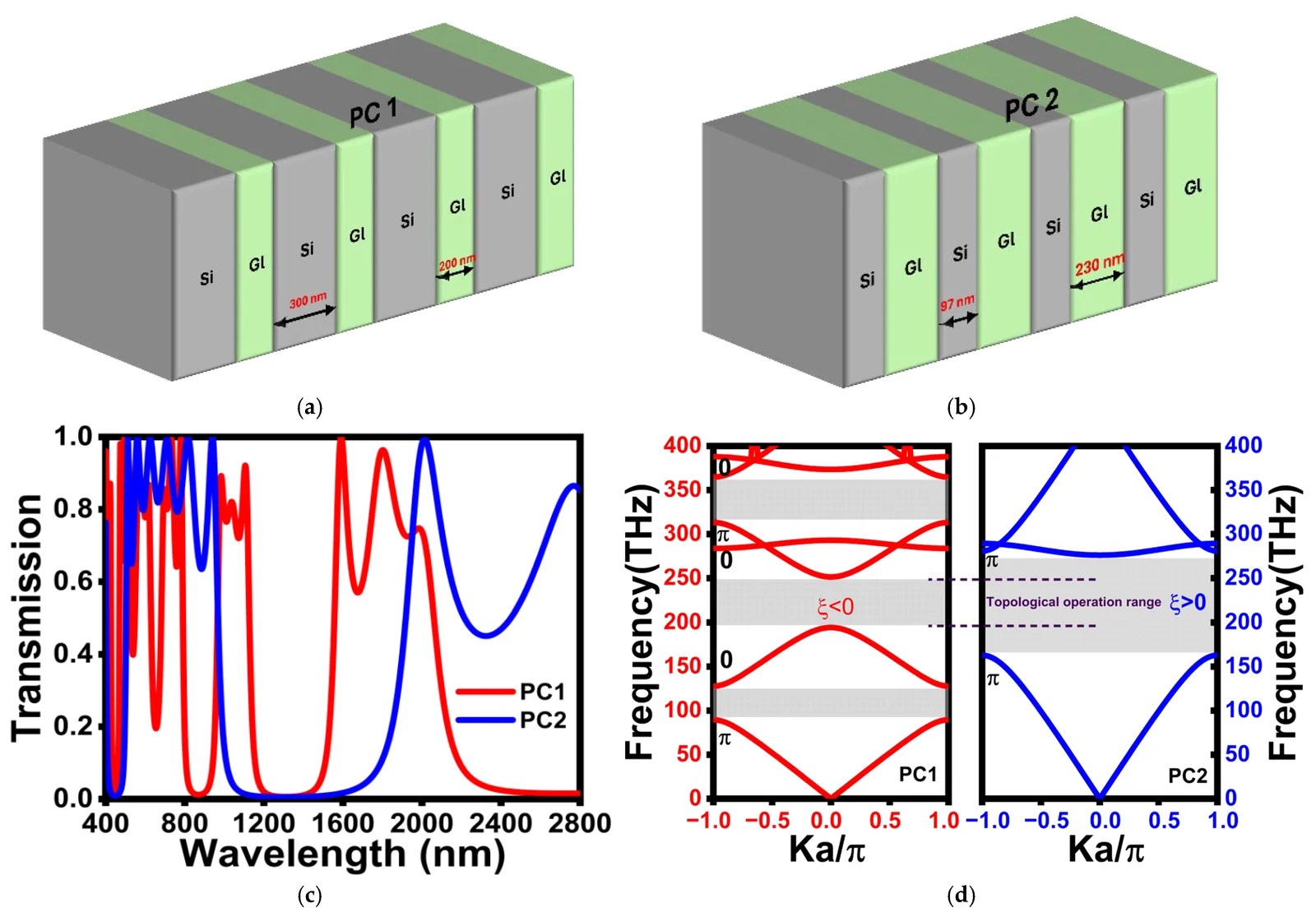
Schematic illustrations of the one-dimensional photonic crystal structures (1DPCs): (**a**) PC1 and (**b**) PC2 with *N* = 4 periods. (**c**) Calculated TE-mode transmission spectra of PC1 and PC2. (**d**) Calculated bulk band structures of PC1 and PC2, with the Zak phases of the relevant bands indicated by 0 and π. The shaded region marks the common operating band gap, corresponding to the second band gap of PC1 and the first band gap of PC2.

The proposed topological mirror heterostructure is initially constructed from two 1DPCs, denoted PC1 and PC2, as illustrated in Figure 1a and Figure 1b, respectively. PC1 consists of four alternating layers (*N* = 4) of silicon (Si) and glycerin (Gl), with layer thicknesses of *t_1Si_* = 300 nm and *t_1Gl_* = 200 nm, respectively. The optical response of the structure is investigated for the transverse electric (TE) polarization using the FEM implemented in COMSOL Multiphysics 6.2. The numerical model has been clarified to explicitly state that the simulations were performed using the real parts of the refractive indices of silicon and glycerin, neglecting material absorption. This assumption was adopted to investigate the idealized resonance characteristics of the proposed structure and isolate the effect of glycerin’s thermo-optic response. The refractive indices are taken as n_Si_ = 3.48 for silicon [32,33], while the refractive index of glycerin is temperature-dependent and expressed as [31] *n_Gl_* = *n_o_* − *α*(*T* − *T_o_*), where n_o_ = 1.4707 is the refractive index at the reference temperature T_o_ = 20 °C, and α = 2.95 × 10^−4^ °C^−1^, which is the thermo-optic coefficient of glycerin.

Glycerin and glycerol are commonly used names for the same chemical compound, C_3_H_8_O_3_; the term “glycerin” is used consistently throughout the present work. To provide a more realistic physical context, the near-infrared optical properties of silicon and glycerin were presented using published datasets from the refractiveindex.info database [34]. At approximately 1380 nm, the optical constant data reported by Franta et al. [33] yield an extinction coefficient of 8.56 × 10^−9^ for crystalline silicon at 300 K. At the same wavelength, the experimental data of Wang et al. [35] give an extinction coefficient of approximately 1.70 × 10^−5^ for glycerin at 22 °C. These values indicate limited absorption during a single passage through the thin constituent layers and provide useful context for the adopted real-refractive-index approximation. Several previous studies have employed simplified optical models neglecting material absorption to investigate silicon-based photonic crystal sensors, demonstrating the roles of refractive-index contrast, field localization, and refractive-index engineering in achieving high-*Q* resonances and enhanced sensing performance [36,37,38,39,40]. Similar modeling approaches have also been applied to near-infrared sensing and localized resonances in photonic crystal structures [41,42]. Overall, these studies provide a solid basis for lossless models to investigate idealized optical responses. Accordingly, the glycerin layer is assumed to be anhydrous, compositionally uniform, and geometrically constrained between mechanically rigid interfaces. Its thickness is therefore maintained at the designed value throughout the investigated temperature range, while the thermal response is represented by the temperature dependence of its refractive index. Moisture uptake, volumetric expansion, internal-pressure variation, and mechanically induced deformation of the liquid-filled cavity are not included in the current index-based model. The predicted sensitivity should therefore be interpreted as the response of an ideal, dry, and geometrically constrained glycerin layer rather than as a complete multiphysics representation of an experimentally packaged device. The relatively large thermo-optic coefficient of glycerin enables efficient refractive-index modulation with small temperature variations, thereby inducing measurable shifts in the photonic structure’s resonance wavelength [14]. The liquid-state nature of glycerin and its compatibility with infiltration-based fabrication techniques make it particularly suitable for integration within multilayer photonic structures. In the proposed configuration, glycerin contributes not only to thermal tunability but also to enhanced light–matter interaction within the resonant cavity. Figure 1c shows the transmission spectra of PC1 and PC2. The relevant photonic bandgap of PC1 is centered at approximately *λ_c_* = 1350 nm, as determined from its calculated transmission response. The conventional quarter-wave condition does not constrain PC1; rather, the thicknesses specified above are the actual parameters used to obtain its band structure and transmission spectrum. The extracted bandgap center of PC1 was subsequently adopted as the target design wavelength for PC2. For PC2, the silicon and glycerin layer thicknesses were selected according to the quarter-wave optical-thickness condition [43], *n*_2Si_*t*_2Si_ = *n*_2Gl_*t*_2Gl_ = *λ_c_*/4, where *n*_2Si_ and *n*_2Gl_ denote the refractive indices of silicon and glycerin, respectively, and *t*_2Si_ and *t*_2Gl_ are their corresponding thicknesses in PC2. Using *λ_c_* = 1350 nm gives *t_2Si_* = 97 nm and *t_2Gl_* = 230 nm. This design aligns the photonic bandgap of PC2 with the relevant bandgap of PC1, providing the common forbidden-frequency region required for the formation of the localized interface state.

The blue curve in Figure 1c represents the transmission spectrum of PC2. The spectral response reveals that the photonic bandgap of PC2 fully overlaps the first bandgap of PC1, thereby creating the conditions required for localized TESs to form at the interface between the two photonic lattices.

The topological distinction between the two constituent photonic crystals was verified by calculating their bulk band structures and the Zak phases of the relevant photonic bands, as shown in Figure 1d. For an inversion-symmetric one-dimensional photonic crystal, the Zak phase is quantized to 0 or π and can be determined from the symmetry of the band-edge eigenmodes at the center and boundary of the Brillouin zone [44,45,46]. The two bands below the operating gap of PC1 have Zak phases of π and 0, whereas the band below the corresponding gap of PC2 has a Zak phase of *π*. When the cumulative Zak phases are considered together with the respective gap order, the second band gap of PC1 is characterized by ζ < 0, while the first band gap of PC2 is characterized by ζ > 0. These gaps overlap with the shaded frequency interval in Figure 1d. The opposite-gap topological signs confirm a topological mismatch between PC1 and PC2 and, consequently, support the formation of an interface-localized state via the bulk–edge correspondence.

In 1DPC systems, interface states can emerge when two PCs possessing overlapping band gaps exhibit distinct topological properties [47]. The bulk topological characteristics of PC1 and PC2 are examined through the Zak phases of their photonic bands, as illustrated in Figure 1d. For the inversion-symmetric unit cells considered here, the Zak phase of an isolated photonic band is quantized to either 0 or π. The calculated band structures reveal different Zak-phase assignments for the bands bordering the common photonic bandgap of PC1 and PC2, demonstrating that the two photonic crystals belong to distinct bulk topological phases. When these topologically dissimilar photonic crystals are joined, the resulting bulk–boundary correspondence permits the formation of an interface state within the overlapping bandgap.

## 3. Results and Discussion

### 3.1. Topological 1DPC

**Figure 2:**
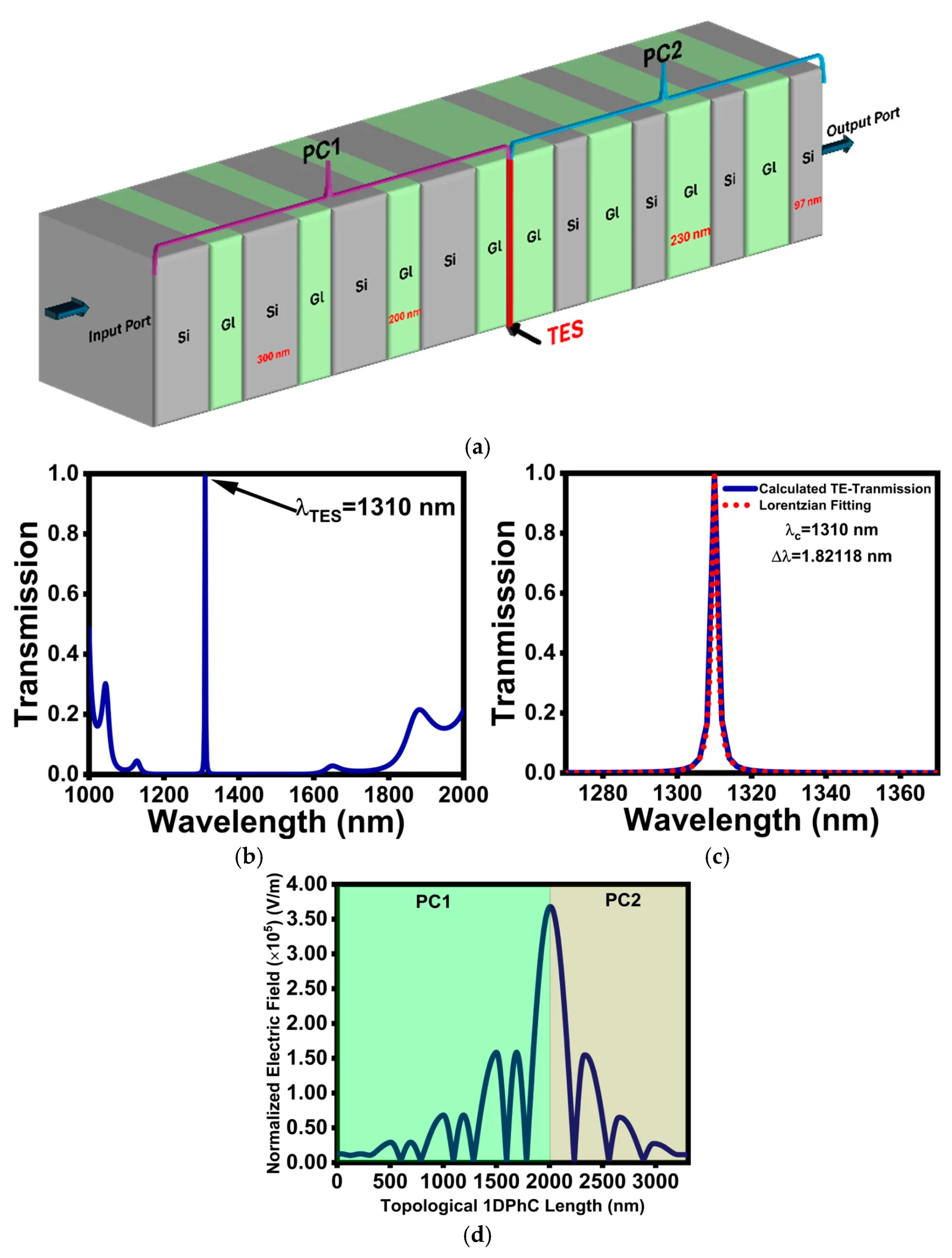
(**a**) Schematic illustration of the topological 1DPC heterostructure formed by connecting PC1 and PC2. (**b**) TE-mode transmission spectrum showing the topological edge state (TES). (**c**) Enlarged view of the TES resonance with Lorentzian fitting. (**d**) Electric-field profile along the propagation direction showing localization of the TES at the heterostructure interface.

Figure 2a illustrates the proposed topological 1DPC obtained by connecting PC1 and PC2 to form a heterostructure interface. The calculated TE-mode transmission spectrum is presented in Figure 2b, where a localized TES appears inside the photonic bandgap near *λ_c_* = 1310 nm. This resonant mode emerges at the interface between the two photonic lattices with distinct topological characteristics. Figure 2c shows an enlarged view of the resonance peak together with its Lorentzian fitting profile [43]. The resonance exhibits near-unity transmission with a narrow full width at half maximum (FWHM) of *Δλ* = 1.82118 nm, indicating strong spectral confinement and localized optical behavior at the heterostructure interface. The quality factor is determined from [12] by *Q* = *λ_c_*/*Δλ*, which yields *Q* = 719.31. The localized nature of the TES is further confirmed by the electric-field profile shown in Figure 2d. Strong field confinement is observed near the interface between PC1 and PC2, indicating the formation of a highly localized resonant mode inside the photonic bandgap. As established by the different Zak-phase assignments presented in Figure 1d, PC1 and PC2 possess distinct bulk topological characteristics across their common photonic bandgap. Consequently, their interface supports the localized topological edge state observed in the transmission spectrum. The corresponding electric-field profile in Figure 2d reaches its maximum near the PC1–PC2 boundary and decays into the surrounding periodic regions, confirming the spatial localization of the interface mode.

### 3.2. Topological Mirror Heterostructure Thermal Sensor

**Figure 3:**
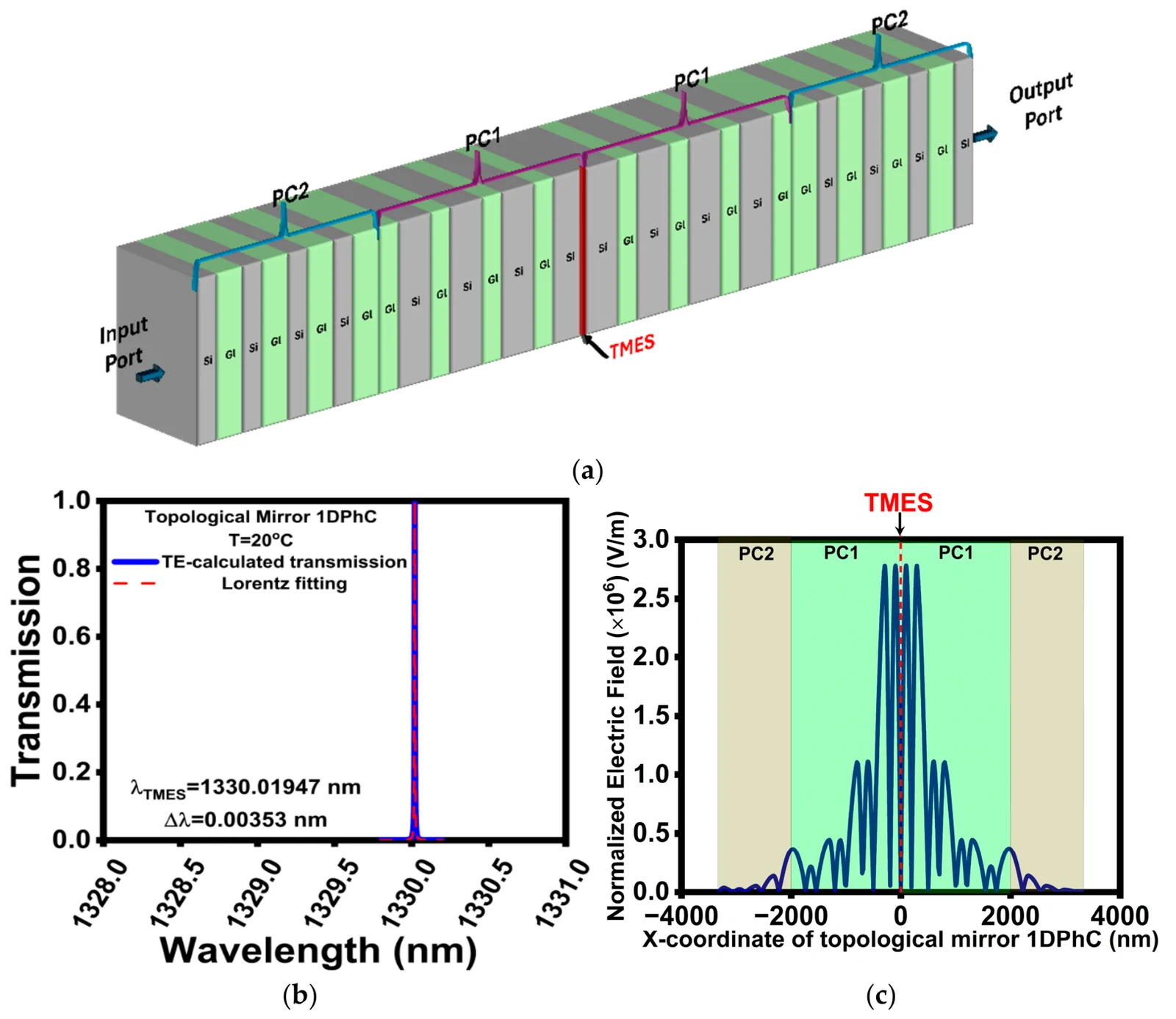
(**a**) A schematic illustration of the topological mirror heterostructure composed of alternating silicon and glycerin layers. (**b**) Enlarged view of the topological mirror edge-state (TMES) resonance centered at *λ_TMES_* = 1330.02 nm with Lorentzian fitting and a narrow linewidth of *Δλ* = 3.53 × 10^−3^ nm. (**c**) Electric-field profile along the propagation direction showing strong localization of the TMES at the mirror interface.

At the ambient temperature *T* = *T_o_* = 20 °C, the refractive index of the glycerin layer is calculated using the above equation, yielding *n_Gl_* =1.4707. The proposed structure, shown in Figure 3a, consists of a mirror-symmetric topological heterostructure formed by alternating silicon (Si) and glycerin (Gl) layers. The mirror configuration enhances optical confinement at the interface region and supports the formation of a strongly localized topological mirror edge state (TMES). The calculated TE-mode transmission spectrum is presented in Figure 3b, where an ultranarrow TMES resonance appears at *λ_TMES_* = 1330.02 nm. The resonance exhibits near-unity transmission with a narrow linewidth of *Δλ* = 3.53 × 10^−3^ nm, corresponding to a quality factor of *Q* = 3.77 × 10^5^. The ultranarrow resonance linewidth indicates strong spectral confinement and high optical selectivity within the photonic bandgap.

The spatial localization of the TMES is further confirmed by the electric-field profile shown in Figure 3c. Strong electric-field confinement is observed near the mirror interface, while the field amplitude decays away from the center of the structure. This behavior confirms the formation of a highly localized resonant mode supported by the topological mirror heterostructure.

The strong localization of the TMES enhances optical confinement within the glycerin-filled cavity, resulting in ultranarrow resonance linewidths and high spectral selectivity. Owing to the negative thermo-optic coefficient of glycerin, temperature variations modify the cavity’s effective refractive index and optical path length, thereby inducing shifts in the resonant wavelength. The combination of strong field localization and thermo-optic tunability enables high-resolution thermal sensing with enhanced *Q*-factor and FOM characteristics.

**Figure 4:**
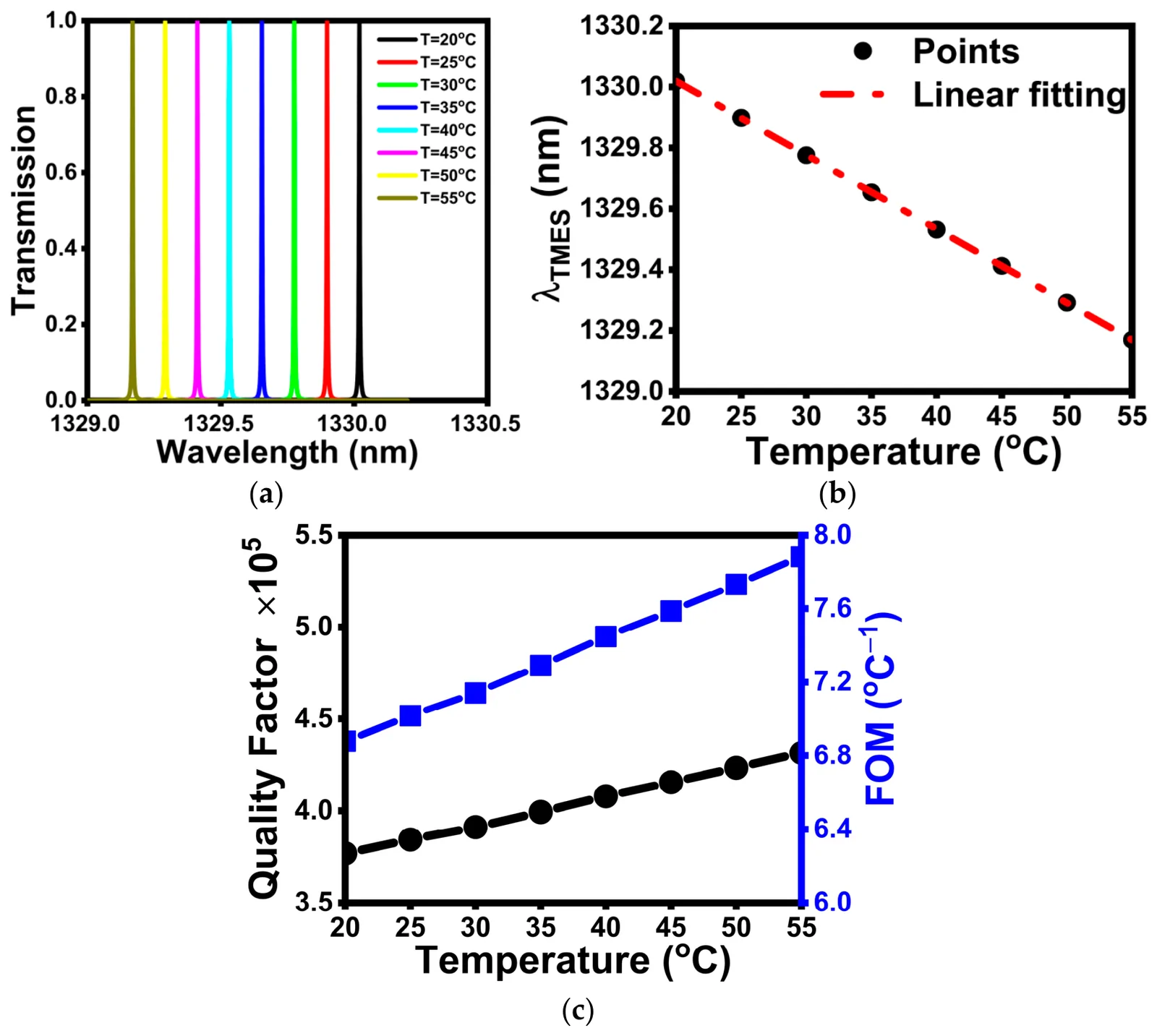
(**a**) Temperature-dependent TE-mode transmission spectra of the topological mirror heterostructure for temperatures ranging from 20 to 55 °C, showing the blueshift of the TMES resonance wavelength as temperature increases. (**b**) Linear variation in the resonant wavelength *λ_TMES_* as a function of temperature. (**c**) Temperature-dependent evolution of the quality factor (*Q*) and the figure of merit (FOM).

The thermal sensing performance of the proposed structure is investigated by varying the temperature from 20 to 55 °C. The corresponding transmission spectra are shown in Figure 4a. As the temperature increases, the TMES resonance undergoes a systematic blueshift toward shorter wavelengths due to the decrease in the refractive index of glycerin. Figure 4b shows the linear variation in the resonance wavelength with temperature. The thermal properties of the initial topological mirror heterostructure, before the introduction of the glycerin defect layer, were studied over the temperature range of 20–55 °C. The resonance undergoes a continuous blueshift due to the negative thermo-optic effect of glycerin as temperature increases. From the linear fit of the resonance wavelength versus temperature as shown in Figure 4b, a thermal sensitivity of *S_T_* = −0.02428 nm/°C, equivalent to 82.3 nm/RIU, has been obtained. This describes the behavior of the initial topological mirror structure. The temperature-dependent evolution of the *Q*-factor and *FOM* is shown in Figure 4c. Both parameters increase gradually with temperature, indicating improved spectral selectivity and resonance sharpness at elevated temperatures. The increase in *FOM* originates primarily from the narrowing of the effective resonance linewidth relative to the resonance shift, highlighting the advantage of strong optical confinement in the proposed topological mirror heterostructure.

### 3.3. Defect-Engineered Topological Mirror Cavity for Enhanced Thermal Sensing

**Figure 5:**
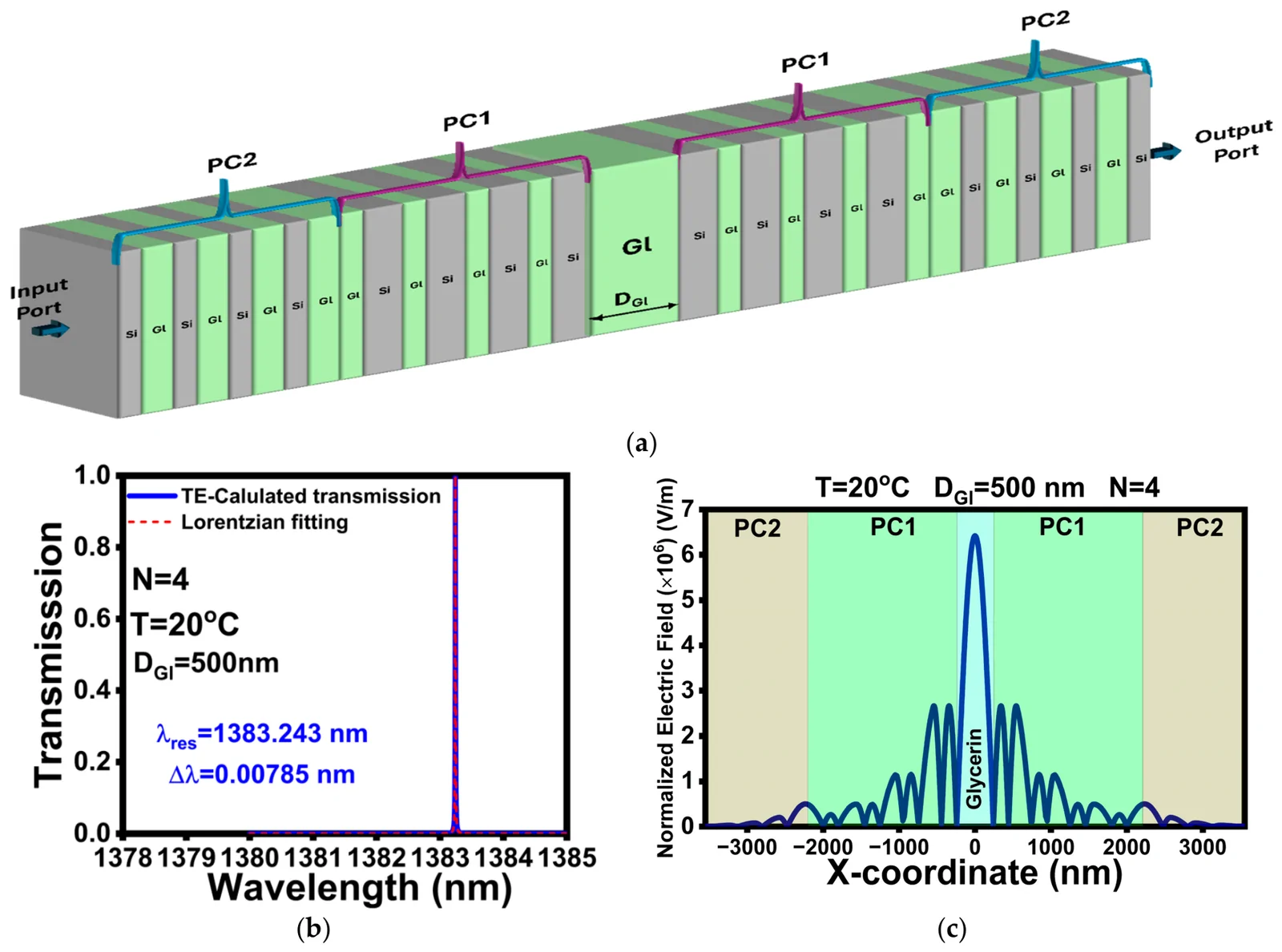
(**a**) Schematic illustration of the defect-engineered topological mirror cavity incorporating a central glycerin defect layer. (**b**) TE-mode transmission spectrum showing the cavity resonance at λ_res_ = 1383.24 nm, with linewidth Δλ = 7.85 × 10^−3^ nm. (**c**) Electric-field profile along the propagation direction demonstrating localization of the resonant mode inside the glycerin-filled cavity region.

Figure 5a illustrates the proposed topological mirror cavity obtained by introducing a central glycerin defect layer between two mirror-symmetric topological heterostructures. Insertion of the central glycerin defect changes both the spatial distribution of the resonant field and the balance between optical confinement and external coupling. As shown in Figure 5b, the initial defect-engineered cavity supports a resonance at approximately 1383.24 nm, with a linewidth of approximately 7.85 × 10^−3^ and a quality factor of *Q* ≈ 1.76 × 10^5^. This quality factor is lower than the value of *Q* ≈ 3.77 × 10^5^ obtained for the mirror heterostructure without the central defect in Figure 3b. Therefore, the glycerin defect should not be interpreted as independently increasing *Q*. Instead, the field distribution in Figure 5c shows that the defect relocates the field maximum into the temperature-sensitive glycerin region and increases the optical-energy overlap with glycerin. This stronger interaction enhances the temperature-induced change in the effective optical path length and, consequently, the thermal sensitivity.

It is important to distinguish the glycerin-induced cavity resonance from the topological interface state formed at a direct boundary between PC1 and PC2. The central glycerin layer does not possess an independent bulk topological invariant; rather, its insertion breaks the local periodicity and produces a Fabry–Pérot-type defect-cavity mode. The surrounding PC1 and PC2 sections retain their distinct bulk topological properties and provide strongly reflecting photonic-crystal boundaries that confine the optical field within the glycerin layer. Thus, the resulting resonance is more accurately described as a defect-engineered cavity mode supported by topological photonic-crystal mirrors, rather than as an independently protected topological interface state. A similar distinction between a Fabry–Pérot cavity mode and a topological edge mode in a one-dimensional topological photonic-crystal heterostructure has been demonstrated by Gao et al. [48].

The physical origin of this resonance differs from that of the coupled topological edge state investigated in our previous work [23]. In the earlier structure, the resonance resulted primarily from coupling between interface-localized topological modes. In the present cavity, the central glycerin defect introduces an additional optical path and relocates the dominant field maximum into the temperature-sensitive region. The topological mirror sections act as highly reflective confinement boundaries, while the defect region governs the thermo-optic perturbation of the resonance. Consequently, varying the defect thickness modifies the field overlap and resonance position, whereas increasing the mirror periodicity mainly suppresses leakage and narrows the resonance. This distinction explains why the quality factor changes much more strongly with mirror length than the thermal sensitivity.

**Figure 6:**
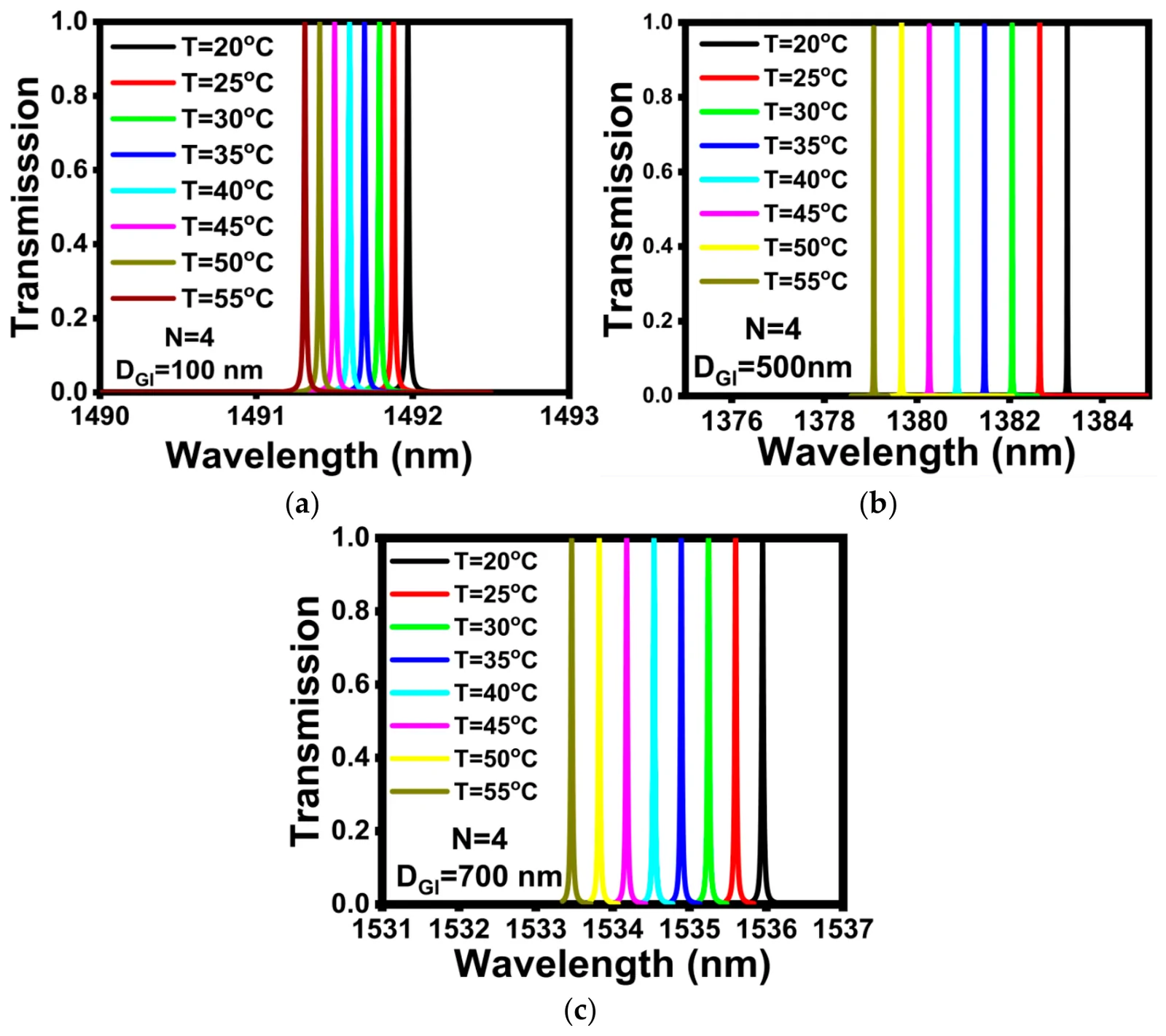
Temperature-dependent TE-mode transmission spectra of the defect-engineered topological mirror cavity for different glycerin defect thicknesses: (**a**) *D_G_*_l_ = 100 nm, (**b**) *D_Gl_* = 500 nm, and (**c**) *D_Gl_* = 700 nm.

Figure 6 presents the temperature-dependent transmission spectra for glycerin defect thicknesses of *D_Gl_* = 100, 500, and 700 nm. For all configurations, increasing temperature produces a systematic blueshift of the resonance wavelength due to the negative thermo-optic coefficient of glycerin. For the smallest defect thickness (*D_Gl_* = 100 nm), the resonance shift remains relatively weak because of the limited interaction volume between the optical mode and the temperature-sensitive glycerin region. Increasing the defect thickness to *D_Gl_* = 500 nm enhances the light–matter interaction, resulting in a more pronounced wavelength shift while preserving a narrow resonance linewidth. For *D_Gl_* = 700 nm, the resonance shift remains observable; however, the spectral confinement is weaker than in the *D_Gl_* = 500 nm configuration. This behavior indicates that excessive defect thickness may reduce the localization efficiency of the cavity mode inside the defect region. Among the investigated configurations, the *D_Gl_* = 500 nm cavity exhibits the best compromise between thermal tunability and spectral confinement, producing the sharpest resonance and the strongest temperature-dependent wavelength shift.

**Figure 7:**
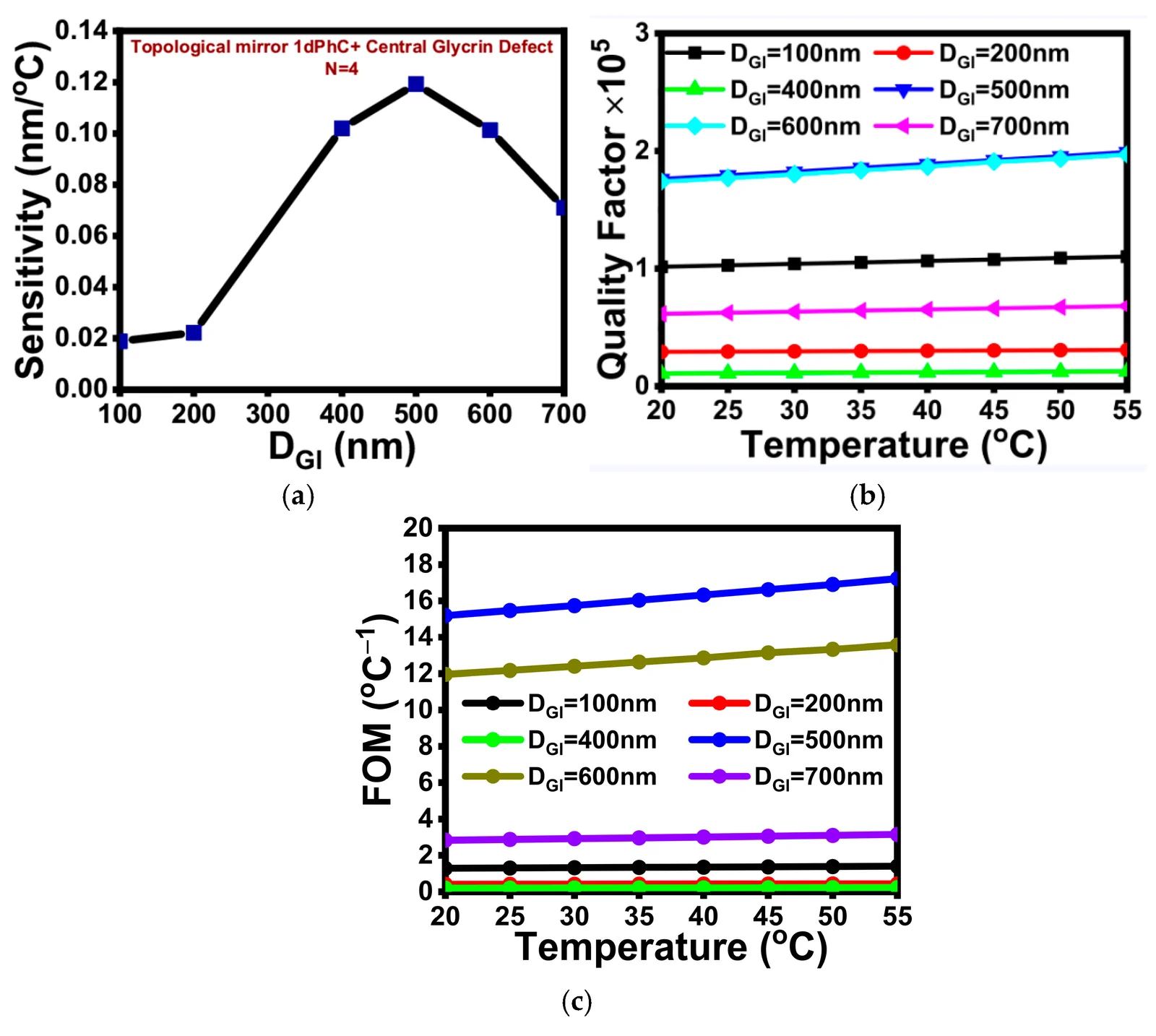
Influence of glycerin defect thickness on the sensing characteristics of the proposed topological mirror cavity: (**a**) thermal sensitivity as a function of defect thickness, (**b**) temperature-dependent quality factor (*Q*), and (**c**) figure of merit (*FOM*) for different glycerin defect thicknesses.

Figure 7a presents the variation in thermal sensitivity with glycerin defect thickness. The sensitivity exhibits a non-monotonic behavior and reaches a maximum value near *D_Gl_* = 500 nm, indicating an optimal balance between optical confinement and thermo-optic interaction. For smaller defects, the interaction length between the optical field and glycerin remains limited, reducing the thermal response. Conversely, excessive defect thickness weakens cavity-mode localization and reduces the efficiency of light–matter interaction, thereby lowering sensitivity.

Figure 7b shows the temperature evolution of the quality factor for different defect thicknesses. The quality factor remains nearly constant over the investigated temperature range, indicating that the cavity retains its spectral confinement characteristics under thermal tuning. The *D_Gl_* = 500 nm configuration exhibits the highest quality factor, exceeding *Q* > 1.7 × 10^5^ throughout the studied temperature range. The corresponding *FOM* values are shown in Figure 7c.

The *D_Gl_* = 500 nm configuration achieves the highest *FOM* value (~17 °C^−1^), owing to the combination of enhanced sensitivity and an ultranarrow resonance linewidth. The increase in *FOM* with temperature indicates improved sensing efficiency at elevated temperatures, while the optimized defect thickness provides enhanced spectral selectivity and strong optical confinement. Consequently, the *D_Gl_* = 500 nm cavity represents the optimal operating condition for high-resolution thermal sensing.

The results in Figure 6 and Figure 7 further distinguish the sensitivity enhancement from the behavior of the quality factor. Figure 6 shows the temperature-dependent displacement of the defect resonance for different glycerin-defect thicknesses, while Figure 7a shows that the thermal sensitivity reaches its maximum near *D_Gl_* = 500 nm. This optimum arises from a balance between field confinement and the volume of glycerin interacting with the resonant mode. By contrast, Figure 7b,c show the corresponding variation, cap *Q*, and *FOM*. These results confirm that a larger wavelength shift does not necessarily require a larger quality factor: the sensitivity is governed mainly by the thermo-optic field overlap, whereas *Q* is determined mainly by the resonance decay and leakage rates.

**Figure 8:**
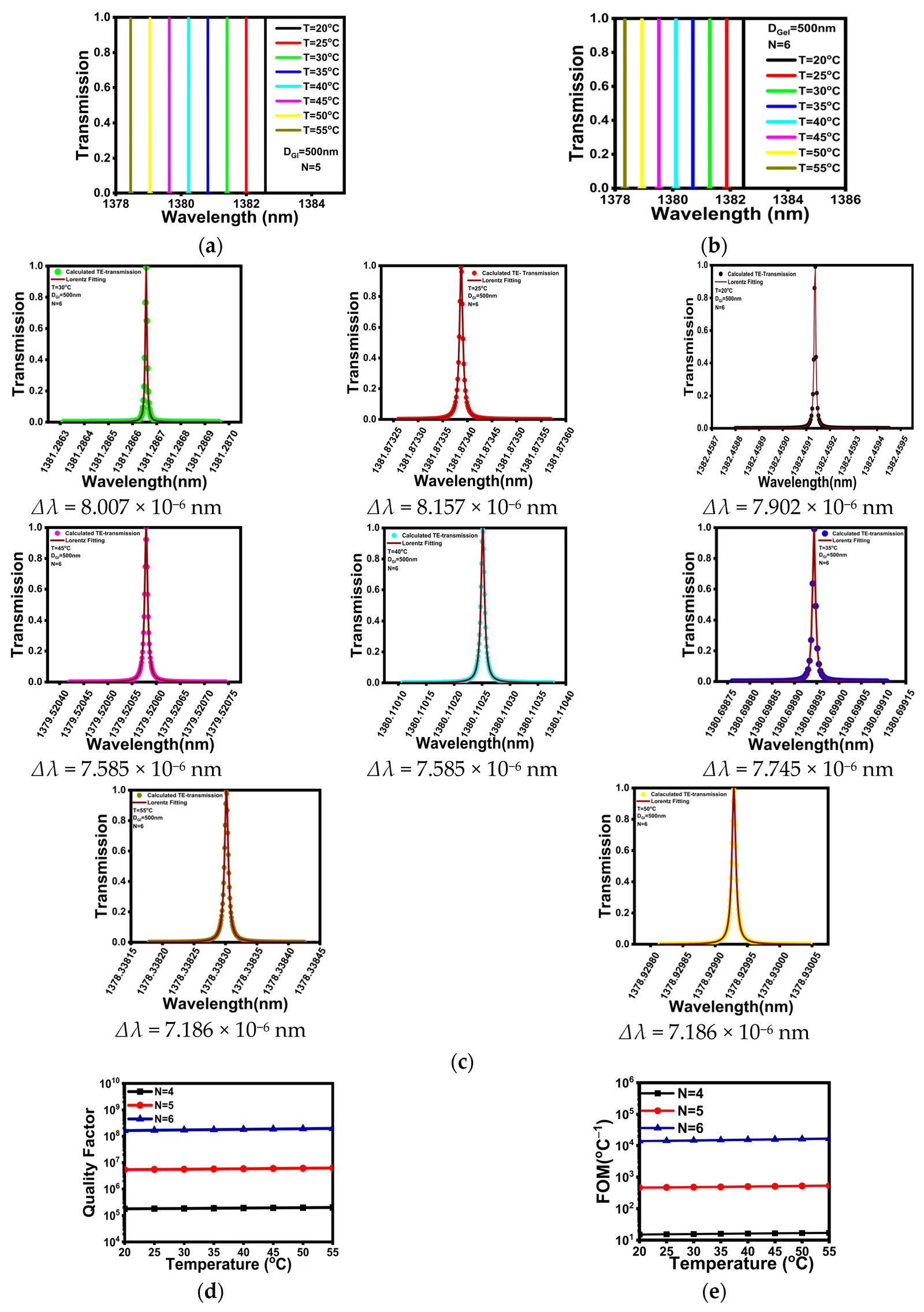
Influence of mirror periodicity on the sensing performance of the defect-engineered topological mirror cavity with *D_Gl_* = 500 nm: (**a**) temperature-dependent TE-mode transmission spectra for *N* = 5, (**b**) temperature-dependent spectra for *N* = 6, (**c**) magnified views of the ultranarrow transmission resonances of the optimized topological mirror cavity (*N* = 6) at selected temperatures within the range of 20−55 °C, showing the high-resolution simulated transmission data and corresponding Lorentzian fits. These panels provide detailed views of the resonance peaks presented in (**b**), (**d**) the quality factor as a function of temperature for different mirror periodicities, and (**e**) the corresponding figure of merit.

To further enhance the sensing performance of the proposed topological mirror cavity, the influence of mirror periodicity was investigated by varying the number of periods in PC1 and PC2 (*N* = 4, 5, and 6), while maintaining the glycerin defect thickness fixed at *D_Gl_* = 500 nm. The temperature-dependent transmission spectra for *N* = 5 and *N* = 6 are presented in Figure 8a and Figure 8b, respectively. For both configurations, increasing temperature produces a systematic blueshift of the resonance wavelength due to the negative thermo-optic coefficient of glycerin.

Because the linewidth of the optimized *N* = 6 resonance is several orders of magnitude smaller than its temperature-induced spectral displacement, it cannot be resolved on the broad wavelength scale used to display the complete temperature-dependent spectra in Figure 8b. To provide a more detailed examination of the ultranarrow resonances observed in Figure 8b, Figure 8c presents magnified transmission spectra of the optimized structure (*N* = 6) at selected temperatures, together with their corresponding Lorentzian fits. A refined wavelength sweep was employed around each resonance to accurately resolve its narrow spectral profile and determine the full width at half maximum (*FWHM*), thereby improving the reliability of the calculated quality factors. At *T* = 35 °C, for example, the spectral curve has an *FWHM* of approximately 7.745 × 10^−6^ nm. The resulting leakage-limited quality factor is *Q = λres*/*FWHM* ≈ 1.77 × 10^8^. Using |S_T_| = 0.1177 nm/°C, the corresponding thermal figure of merit is *FOM* = *|S_T_|*/*FWHM* ≈ 1.52 × 10^4^ °C^−1^.

These values represent predicted numerical upper bounds under the lossless, perfectly periodic conditions considered in the present model. The additional mirror layers strengthen the photonic confinement around the defect region, thereby improving the spectral selectivity of the resonant state. The pronounced increase in *Q* with mirror periodicity originates from the progressive suppression of radiation leakage as the reflectivity of the photonic mirrors increases. This behavior is consistent with previous investigations of high-*Q* photonic-crystal cavities. Nawi et al. [49] reported simulated and experimental quality factors of approximately 7.4 × 10^5^ and 8 × 10^5^, respectively, in a one-dimensional silicon photonic crystal microcavity with *N* = 6 periodic mirror holes. For a more closely related topological configuration, Barvestani [50] investigated a one-dimensional topological heterostructure comprising six unit cells on each side of the interface and demonstrated pronounced resonance narrowing as the periodicity increased, with topological interface-state quality factors on the order of millions. More generally, theoretical studies have shown that optimized lossless photonic crystal nanocavities can support quality factors approaching 10^9^ when radiation leakage is strongly suppressed [51]. These findings support the physical plausibility of the high-*Q* regime obtained for the present *N* = 6 configuration. The substantial increase in the calculated quality factor with mirror periodicity reflects the enhanced optical confinement and reduced resonance leakage achieved by the optimized structure. Under the adopted real-refractive-index approximation, the predicted *Q*-factor approaches 1.8 × 10^8^, and the corresponding *FOM* represents the predicted, leakage-limited response of the cavity. Although the extinction coefficients of silicon and glycerin are relatively small within the investigated near-infrared region, their contribution to resonance broadening may become important when the optical field remains confined for an extended period. The ultranarrow full-width at half maximum (*FWHM*) reported for the optimized *N* = 6 structure is a computationally derived, leakage-constrained linewidth rather than an empirically obtained measurement. Analogous computations of optimal photonic-crystal cavities have predicted radiation-limited quality factors in the range of 10^8^–10^9^ [52,53], reinforcing the theoretical feasibility of significant leakage mitigation in adequately confined architectures. Nonetheless, the current number must be regarded as a theoretical maximum, as experimental linewidths would also encompass material, manufacturing, coupling, and instrumental widening. The influence of mirror periodicity on the quality factor and *FOM* is shown in Figure 8d and Figure 8e, respectively. Increasing the number of periods from *N* = 4 to *N* = 6 increases the reflectivity of the surrounding Bragg mirrors, suppresses leakage from the defect-localized mode, and progressively narrows the resonance. Consequently, the leakage-limited quality factor increases from the order of 10^5^ for *N* = 4 to a numerically calculated value approaching 1.77 × 10^8^ for *N* = 6, as shown in Figure 8d. The corresponding linewidth reduction produces the increase in *FOM* shown in Figure 8e. The substantial increase in the calculated *Q*-factor with mirror periodicity, as shown in Figure 8d, reflects the enhanced optical confinement and reduced resonance leakage as the photonic mirrors become more reflective. Under the adopted lossless approximation, the predicted *Q*-factor approaches 1.8 × 10^8^, and the corresponding *FOM* represents the predicted, leakage-limited sensing performance of the optimized structure (*N* = 6). Although the reported extinction coefficients of silicon and glycerin are relatively small within the investigated near-infrared region, material absorption may significantly affect such extremely high-*Q* resonances by broadening the linewidth and reducing the resonance lifetime. In practical devices, additional losses associated with interface imperfections and fabrication deviations may further reduce the achievable *Q*-factor and FOM. Therefore, the reported values should be interpreted as idealized numerical predictions and experimentally achievable values [35,54,55].

To independently assess the high-quality factors extracted from the transmission spectra, a complementary eigenfrequency analysis was performed for the optimized topological mirror cavity. Following the established eigenfrequency-based formulation [56,57]:

(1) $$Q_{\mathrm{Eigen}.}=\frac{\mathrm{real}(f_{r})}{2\times \mathrm{imag}(f_{r})}$$

where *fr* is the complex eigenfrequency corresponding to the resonance wavelength *λ_res_*. The calculations employed a user-controlled triangular mesh with targeted local refinement near the central glycerin cavity and neighboring photonic-crystal interfaces. As summarized in Table 1, the quality factors obtained from the eigenfrequency analysis agree closely with those extracted from transmission-based Lorentzian fitting over the temperature range of 20–55 °C, with relative differences below 0.49%. This agreement provides additional numerical support for the reported high-*Q* response of the optimized structure.

To further evaluate the sensing capabilities of the optimized topological mirror cavity (*N* = 6, *D_Gl_* = 500 nm), additional performance parameters were calculated, including the spectral shift-to-linewidth ratio (*SNR*), spectral resolution (*Rs*), and estimated temperature detection limit (*DL_T_*). Following the approach reported by Zaky et al. [58], these parameters were evaluated using:

(2) $$\mathrm{SNR}\;=\frac{|\Delta \mathrm{\lambda res}|\;}{\mathrm{FWHM}}$$

(3) $$R_{s}=\frac{2\times \mathrm{FWHM}}{3{\left(\mathrm{SNR}\right)}^{\frac{1}{4}}}$$

(4) $${\mathrm{DL}}_{T}=\frac{R_{s}}{\left|S_{T}\right|}$$

where *Δλ_res_* is the resonance wavelength displacement relative to the reference temperature of 20 °C, *FWHM* denotes the resonance linewidth, and *S_T_* represents the thermal sensitivity. Over the temperature range of 25–55 °C, the calculated average values of *SNR*, spectral resolution, and temperature detection limit were approximately 3.1908 × 10^5^, 2.2873 × 10^−7^ nm, and 1.9427 × 10^−6^ °C, respectively. The reference temperature was excluded from the averaging because the resonance displacement relative to itself is zero. These results characterize the idealized spectral sensing performance of the optimized structure, while the experimentally achievable detection limit would additionally depend on measurement noise and instrumental resolution.

In contrast, the thermal sensitivities summarized in Table 2 vary only slightly with the number of mirror periods. For *N* = 4, 5, and 6, the calculated signed sensitivities are −0.1193, −0.1180, and −0.1177 nm/°C, respectively. This weak dependence on *N* confirms that mirror periodicity primarily controls the resonance leakage, linewidth, quality factor, and *FOM*, whereas the thermal sensitivity is governed mainly by the interaction between the localized optical field and the central glycerin defect. Although the thermal sensitivity varies only slightly with mirror periodicity (Table 2), increasing *N* substantially improves the spectral selectivity, *Q*-factor, and *FOM*. Therefore, the *N* = 6 configuration provides optimal cavity performance for high-resolution thermal sensing. Table 2 shows that the thermal sensitivity varies only slightly with the number of mirror periods. This indicates that the thermal response is governed primarily by the glycerin-defect region rather than by the mirror length. Since the resonance shift originates from thermo-optic modulation within the defect layer, variations in the mirror periodicity primarily affect optical confinement rather than the sensitivity itself [43,59,60]. The quality factors in Figure 8d are leakage-limited numerical values calculated under the assumptions of lossless materials, perfectly smooth interfaces, and exact structural periodicity.

The main contribution of the present study is the design and systematic optimization of a glycerin-filled defect cavity, which is embedded into a topological mirror heterostructure. Although the topological mirror concept has been explored in a previously reported thermal sensor [23], the present configuration introduces a distinct sensing mechanism based on a central glycerin defect surrounded by topological photonic mirrors. This arrangement allows the thermo-optic interaction within the defect region and the optical confinement provided by the mirrors to be adjusted through two structural parameters: the glycerin defect thickness and the mirror periodicity. The successive design stages demonstrate the importance of optimizing these parameters together. Introducing the glycerin defect initially reduces the calculated quality factor from 3.77 × 10^5^ to 1.76 × 10^5^. However, this reduction occurs in an intermediate configuration before optimization. By adjusting the defect thickness and increasing the mirror periodicity, the final optimized structure achieves a calculated quality factor of approximately 1.8 × 10^8^, and a figure of merit of approximately 1.53 × 10^4^ °C^−1^, while maintaining a thermal sensitivity of −0.1177 nm/°C. For comparison, the previously reported structure exhibited a quality factor of approximately 1.0 × 10^7^, a figure of merit of 1002.48 °C^−1^, and a thermal sensitivity of −0.12317 nm/°C. The calculated quality factor of the present optimized structure is therefore approximately 18 times that of the previous design, while its figure of merit is approximately 15 times as high. Although the thermal sensitivity is slightly lower, the optimized configuration offers substantially improved spectral selectivity under the predicted modeling assumptions. These results highlight the complementary roles of the glycerin defect in providing thermo-optic interaction and the surrounding topological mirrors in controlling resonance leakage. A broader comparison with previously reported one-dimensional photonic crystal thermal sensors is presented in Table 3. Although some of the reported structures exhibit higher thermal sensitivity, the proposed optimized structure combines a temperature-responsive glycerin defect with strong optical confinement to achieve a narrow-calculated resonance linewidth and high spectral selectivity. The comparison highlights the importance of accounting for the quality factor, the figure of merit, and thermal sensitivity when evaluating the theoretical performance of photonic crystal sensors.

To further clarify the underlying sensing mechanisms, Table 3 distinguishes the previously reported structures by their cavity configurations and resonant characteristics. Conventional photonic-crystal sensors generally rely on periodic Bragg-type multilayers or defect-induced resonances, whereas topological configurations employ interface states associated with distinct photonic band properties. The proposed structure combines a glycerin-filled Fabry–Pérot-type defect cavity with topologically distinct photonic-crystal mirrors. To provide a platform-consistent assessment, Table 3 compares the proposed design specifically with previously reported one-dimensional photonic-crystal sensors employing related defect- or interface-state sensing mechanisms. Table 3 intentionally compares the proposed structure with other one-dimensional photonic-crystal sensors because they share related multilayer geometries, normal-incidence interrogation, and defect- or interface-state sensing mechanisms. However, the broader ultrahigh-Q landscape is dominated by integrated resonators, including microrings, microdisks, spiral resonators, and two-dimensional photonic-crystal nanocavities. Puckett et al. demonstrated a planar integrated Si_3_N_4_ all-waveguide ring resonator with an experimentally measured intrinsic quality factor of 4.22 × 10^8^, achieved through substantial suppression of absorption and scattering losses [66]. Tanaka et al. theoretically designed a two-dimensional photonic-crystal nanocavity with a quality factor approaching 10^9^ by minimizing out-of-plane radiation leakage [51]. Similarly, Vasco and Savona predicted *Q* = 4.33 × 10^7^ for an optimized silica-encapsulated silicon L3 cavity, although their disorder analysis indicated that realistic structural imperfections could reduce the quality factor to the order of 2 × 10^6^ [67]. Accordingly, ultrahigh *Q* is not unique to the present structure. The distinction of the proposed design lies in combining a one-dimensional topological interface state with a glycerin-filled thermal-sensing cavity and normal-incidence spectral interrogation. In microring and microdisk resonators, light is predominantly confined by total internal reflection and is commonly introduced via an evanescently coupled bus waveguide, making the measured response dependent on both intrinsic resonator loss and coupling conditions. By contrast, the present multilayer structure is interrogated through axial transmission, and the glycerin region forms part of the localized cavity mode, providing direct thermo-optic modulation of the resonance. The proposed system, therefore, targets a different balance among optical confinement, analyte–field overlap, thermal sensitivity, structural simplicity, and interrogation geometry. Its calculated Q ≈ 1.8 × 10^8^ should be interpreted as a predicted, leakage-limited numerical upper bound rather than as a claim of superiority over experimentally established integrated ultrahigh-Q resonators.

### 3.4. Fabrication Tolerance and Robustness Analysis

To evaluate the persistence of the optimized resonance under representative fabrication deviations, two complementary tolerance analyses were performed for the *N* = 6 structure at *T* = 35 °C. In the first analysis, the thicknesses of all periodic silicon and glycerin layers were varied simultaneously by −3%, −1%, +1%, and +3%, while the central glycerin-defect thickness was fixed at its nominal value of 500 nm.

This test represents a correlated systematic deviation that may arise from calibration or process-dependent fabrication errors. In the second analysis, the periodic layers were retained at nominal dimensions, while the central defect thickness was independently varied over the same percentage range. This test evaluates the resonance’s response to a localized dimensional perturbation within the optically active cavity. The resonance wavelength, peak transmission, *FWHM*, and *Q*- factor were recalculated for each configuration, as summarized in Table 4 and Table 5. As shown in Table 4, the ultranarrow resonance remains identifiable throughout the investigated range of correlated periodic-layer deviations. Relative to the nominal resonance at 1380.70 nm, negative thickness deviations shift the resonance toward shorter wavelengths, whereas positive deviations shift it toward longer wavelengths. The quality factor remains of the order of 10^8^, ranging from approximately 1.42 × 10^8^ to 1.68 × 10^8^, compared with the nominal value of about 1.8 × 10^8^. However, the reduction in peak transmission to 0.148 for the +3% case demonstrates that the coupling efficiency can be strongly affected even when the resonance itself survives.

The independent defect-thickness variations in Table 5 similarly preserve a distinct ultranarrow resonance across the complete tolerance range. The quality factor remains between approximately 1.36 × 10^8^ and 1.73 × 10^8^, while the resonant wavelength shifts with the modified optical path length of the glycerin cavity. The lower peak transmission observed for the −3% defect variation indicates that the resonance amplitude is more sensitive to certain dimensional deviations than to the mode’s existence. To further investigate the robustness of the optimized structure, two independent tests of local thickness perturbations were conducted at 20 °C. The layer sequence of the proposed structure is [(Si/Gl)_6_(Gl/Si)_6_/Gl/(Si/Gl)_6_(Gl/Si)_6_] as specified in Table 3. In the first test, the thickness position 4 in PC1, with a nominal thickness of 200 nm, varied by 3%, i.e., to 194 and 206 nm. In the second test, the silicon layer at position 4 in PC2, nominally 97 nm thick, was independently varied by ±3%, corresponding to thicknesses of 94.09 and 99.91 nm. All other structural parameters were kept at their nominal values in each test. The localized resonance was still observed in both cases, and the *Q*-factor remained in the range of approximately 1.64 × 10^8^–1.67 × 10^8^, compared to approximately 1.75 × 10^8^ for the nominal configuration. The results provide a first estimate of the optimized structure’s response to independent, localized deviations in layer thickness. Overall, deviations near ±3% generally preserve both an ultranarrow linewidth and comparatively high transmission, whereas larger deviations may substantially alter the spectral position and coupling strength. The robustness discussed here, therefore, refers specifically to the survival of the localized resonance under bounded structural perturbations and should not be interpreted as complete immunity of all sensing parameters to fabrication errors. The present work constitutes a deterministic tolerance assessment; a fabrication tolerance analysis that accounts for independent deviations in individual layers would be required to characterize the full probability distribution of device performance under uncorrelated fabrication disorder.

## 4. Fabrication and Practical Implementation

The proposed topological mirror heterostructure can be practically realized using established photonic microfabrication techniques, including photolithography, dry etching, and multilayer deposition methods [68,69,70,71,72]. The one-dimensional silicon multilayer configuration provides a straightforward platform for fabrication using conventional optical lithography processes [73,74]. Following the formation of the silicon framework, glycerin can be introduced into the designated cavity and defect regions. Owing to its liquid state and compatibility with optofluidic integration, glycerin can be selectively delivered into the photonic structure via capillary-assisted infiltration or optofluidic microsystems (OFMs) [75,76]. A possible experimental implementation of the proposed structure could employ an in-plane silicon optofluidic platform. The PC1 and PC2 mirror sections, the central defect cavity, and the associated fluidic channels could be patterned on a suitable silicon substrate using lithographic processing followed by reactive-ion or deep-reactive-ion etching. Focused-ion-beam milling could also be employed to define the smallest photonic crystal features. Goyal et al. experimentally demonstrated the fabrication of photonic crystal cavity devices by combining optical lithography and reactive ion etching with focused ion beam processing [73].

After fabrication of the silicon framework, a transparent cover containing fluidic inlet and outlet ports could be aligned and bonded to the patterned substrate to form a closed optofluidic cell. Degassed glycerin could then be introduced into the periodic gaps and central defect cavity through capillary-assisted or controlled-pressure filling. Perova et al. experimentally fabricated a periodically grooved silicon photonic structure, infiltrated its grooves with a functional liquid, and characterized its temperature-dependent optical response [76]. In addition, Surdo et al. fabricated an all-silicon optofluidic microsystem integrating high-aspect-ratio one-dimensional photonic crystals and optical cavities with liquid reservoirs, infiltration channels, and optical-access features [75]. These experimental studies support the feasibility of fabricating the silicon framework and subsequently filling the periodic gaps and central cavity with glycerin. The completed filling should be confirmed visually before the inlet, and outlet ports are hermetically sealed to minimize moisture exchange, evaporation, contamination, leakage, and bubble formation.

The optimized mirror length of *N* = 6 is also within an experimentally demonstrated fabrication range. Muszyński et al. fabricated and optically characterized a liquid-crystal microcavity enclosed by two distributed Bragg reflectors, each composed of six SiO_2_/TiO_2_ pairs [77]. Similarly, Chiasera et al. fabricated an active central defect layer between two six-pair SiO_2_/TiO_2_ Bragg mirrors using radio-frequency sputtering, and the corresponding near-infrared photonic stopband and defect-cavity resonance were experimentally observed [78]. Although these experimental devices employ different constituent materials, they demonstrate that a central liquid or solid-defect cavity bounded by six-period one-dimensional mirrors can be constructed in practice. Therefore, the selected value *N* = 6 is reasonable from the standpoint of structural fabrication.

The proposed structure is compatible with well-known microfabrication techniques, such as lithographic patterning, dry etching, and multilayer processing, from a fabrication standpoint, and the glycerin-filled cavity can be fabricated using established optofluidic integration techniques [68,69,70,71,72,73,74,75,76]. Experimental demonstrations of the fabrication of silicon-based photonic crystal structures and the integration of liquid-filled regions have been reported previously [73,75,76], thereby demonstrating the practical feasibility of the proposed architecture. However, surface roughness and structural defects can lead to additional optical scattering and modification of the resonant characteristics, as observed in studies of photonic crystal fabrication disorder [54]. Fluctuations in the optical properties of the constituent materials may also affect the resonance conditions and spectral confinement.

Nevertheless, layer-thickness deviations, interface or sidewall roughness, incomplete glycerin filling, trapped bubbles, bonding-induced stress, and material absorption would broaden the measured resonance and reduce the experimentally attainable quality factor relative to the ideal leakage-limited numerical prediction.

An important consideration in the experimental realization of the proposed structure is glycerin’s hygroscopic nature. Exposure to ambient air can lead to absorption of water vapor, while the refractive index of glycerin–water mixtures varies systematically with composition. Uncontrolled moisture uptake may therefore change the baseline refractive index of the defect layer and modify its effective temperature-dependent optical response. Accurate optical characterization of glycerin consequently requires careful prevention of water contamination [79]. For practical fabrication, anhydrous, high-purity glycerin should be handled under controlled, low-humidity conditions, with exposure to ambient air minimized during transfer and filling.

The introduction of glycerin into confined optical regions has been demonstrated experimentally. In particular, vacuum-pressure infiltration has been used to fill the air channels of a microstructured optical fiber with glycerin [80]. A similar vacuum-assisted or controlled capillary-filling approach could be adapted to introduce glycerin into the defect region of the proposed heterostructure while reducing the formation of trapped air bubbles. After filling, the inlet should be closed immediately, and the liquid-containing region hermetically encapsulated to preserve its composition. The feasibility of using sealed glycerin-containing liquids in optical temperature sensors has also been demonstrated experimentally [81]. Thermal expansion must additionally be considered because the density and volume of glycerin vary with temperatures [82]. However, the volumetric expansion of the liquid does not necessarily correspond to an increase in the optical defect thickness, which is defined by the separation between the silicon interfaces along the direction of light propagation. In the present theoretical model, this separation is maintained at the designed value of 500 nm throughout the investigated temperature range (20–55 °C). In a practical implementation, the resulting geometrical stability would depend on the mechanical rigidity of the cavity-defining interfaces and the use of an appropriate spacer. If the liquid were confined within a completely sealed, noncompliant volume, thermal expansion could increase the internal pressure, whereas mechanical compliance could lead to cavity deformation and a corresponding change in the optical path length. A possible approach to accommodating liquid expansion is to integrate a sealed microfluidic connection with an external compensation reservoir. The experimental feasibility of integrating one-dimensional silicon photonic crystals with microfluidic channels and reservoirs has been demonstrated [75], while related silicon-based optical microcavities incorporating flowing liquids have also been reported [83]. Such an arrangement could accommodate thermally induced changes in glycerin volume outside the optically active region, provided that the cavity-defining interfaces remain mechanically stable. Controlled filling and hermetic encapsulation would be essential to limit moisture absorption and preserve the assumed anhydrous composition. The effectiveness of these measures in maintaining spectral stability would require experimental verification and calibration over the intended operating temperature range.

The fabricated response can also be affected by spatial differences in the refractive indices of silicon and glycerin arising from residual temperature differences, partial glycerin filling, contamination, or even changes in glycerin composition due to moisture absorption. As indicated by experimental data on refractive index, glycerin is a hygroscopic liquid with wavelength- and temperature-dependent optical properties; hence, protection against water contamination is vital to ensure the reproducibility of the thermo-optic response [79]. Hence, glycerin-filled zones should be filled with clean, degassed liquid and sealed to maintain their composition and prevent air bubbles from forming. During thermal characterization, the device should be mounted on a broad temperature-controlled stage and heated gradually until thermal equilibrium is established before each optical spectrum is recorded. The use of a broad heater, stable thermal contact, and an enclosed measurement environment can reduce longitudinal and transverse temperature gradients across the active photonic region. Coupled thermal–optical simulations have shown that heater geometry, thermal contacts, and heat dissipation through the surrounding medium govern the temperature distribution and thermo-optic response of photonic-crystal devices [84]. Experimentally, the temperature can be monitored at more than one position near the structure, while spatially resolved thermometry may be employed when a direct evaluation of the residual gradient is required. The feasibility of accurately measuring longitudinal temperature distributions in optical structures has been demonstrated by [85]. These measures would limit unintended refractive-index nonuniformity and improve correspondence between the experimental device and the uniform-temperature approximation adopted in the present optical model.

## 5. Conclusions

The present work proposes a defect-engineered topological mirror heterostructure based on alternating layers of silicon and glycerin for high-resolution thermal sensing applications. The proposed structure strongly supports localized topological mirror edge states, enabling enhanced optical confinement and ultranarrow resonant modes within the photonic bandgap. The optimized structure exhibits a calculated thermal sensitivity of −0.1177 nm/°C, with a corresponding figure of merit of approximately 1.53 × 10^4^ °C^−1^ and a quality factor approaching 1.8 × 10^8^ under the adopted real-refractive-index approximation. These values reflect the strong optical confinement and narrow resonance linewidth predicted for the proposed design. The enhanced performance of the proposed sensor stems from the strong localization of the topological mirror edge state within the glycerin-filled cavity, enabling optical confinement, ultranarrow resonance linewidths, and high spectral selectivity. Combined with the strong thermo-optic response of glycerin, the proposed structure achieves high-resolution thermal sensing with ultrahigh quality-factor and figure-of-merit characteristics. The results demonstrate the potential of combining glycerin thermo-optic modulation with topological photonic confinement to enable compact, integrated, high-resolution thermal sensing platforms.

## Figures and Tables

**Table 1:** Comparison of resonance wavelengths, resonance frequencies, and quality factors obtained from transmission-based Lorentzian fitting and complex eigenfrequency analysis for the optimized topological mirror cavity (*N* = 6, *D_Gl_* = 500 nm) over the temperature range of 20–55 °C, together with their relative differences.

*T* (°C)	Transmission Solving			Eigenfrequency Solver			Relative Difference (%)		
	*λ_TMES_* (nm)	*fr* (Hz)	*Q_Trans._*	*fr* (Hz)	*λ_TMES_* (nm)	*Q_Eigen._*	Δ*λ_TMES_* (%)	*Δf* (%)	*ΔQ* (%)
20	1382.4593	2.1700 × 10^14^	1.6354 × 10^8^	2.1689 × 10^14^	1383.1861	1.6275 × 10^8^	0.0526	0.0525	0.4862
25	1381.8736	2.1710 × 10^14^	1.6797 × 10^8^	2.1695 × 10^14^	1382.8300	1.6715 × 10^8^	0.0692	0.0692	0.4862
30	1381.2868	2.1719 × 10^14^	1.725 × 10^8^	2.1704 × 10^14^	1382.2429	1.7167 × 10^8^	0.0692	0.0692	0.4847
35	1380.6991	2.1728 × 10^14^	1.7716 × 10^8^	2.1713 × 10^14^	1381.6548	1.7630 × 10^8^	0.0692	0.0692	0.4857
40	1380.1104	2.1737 × 10^14^	1.8194 × 10^8^	2.1722 × 10^14^	1381.0657	1.8106 × 10^8^	0.0692	0.0692	0.4837
45	1379.5208	2.1747 × 10^14^	1.8685 × 10^8^	2.1732 × 10^14^	1380.4756	1.8594 × 10^8^	0.0692	0.0692	0.4841
50	1378.9301	2.1756 × 0^14^	1.9188 × 10^8^	2.1741 × 10^14^	1379.8845	1.9095 × 10^8^	0.0692	0.0692	0.4804
55	1378.3385	2.1765 × 10^14^	1.9705 × 10^8^	2.1750 × 10^14^	1379.2925	1.9610 × 10^8^	0.0692	0.0692	0.4862

**Table 2:** Effect of the number of periods on the Sensitivity of the proposed topological mirror 1DPC thermal sensor.

*N*	*S_T_* (nm/°C)	*S* (nm/RIU)
4	−0.1193	404.5
5	−0.1180	400
6	−0.1177	399.1

**Table 3:** Comparison of the proposed thermal sensor with previously reported one-dimensional photonic-crystal sensors.

Reference	Structure-Resonance Mechanism/Cavity Type	*S_T_* (nm/°C)	*Q.F*	*FOM* (°C^−1^)	Temp. Range
[12]	[(Si)(air/Si)^3^(LC)(Si/air)^3^(Si)] (Conventional Bragg defect cavity)	0.328	4840	N.A	15–55 °C
[23]	[(Si/Silica)^6^(Silica/Si)^6^/Silica/(Si/Silica)^6^(Silica/Si)^6^] (Coupled topological interface states)	0.12317	~10^7^	1002.48	0–50 °C
[28]	[(TiO_2_)^6^ SiO_2_ (SiO_2_)^6^] (Conventional defect-mode cavity)	0.0037	N.A	N.A	0–300 °C
[15]	[(Si/air)^5^ Si (air/Si)^5^] (Conventional Bragg defect cavity)	0.064	N.A	N.A	100–700 K
[30]	(Si/SiO_2_/PS)^N^ with N = 10 (Annular photonic crystal with liquid-crystal sensing)	0.224	N.A	20.36	15–55 °C
[61]	[Si/polymer/SiO_2_] (Conventional ternary photonic crystal)	0.380	N.A	N.A	25–100 °C
[62]	[(Al_2_O_3_)^5^ Toluene (TiO_2_)^5^] (Defect/interface-state cavity)	0.0887	471.4	N.A	−20–70 °C
[63]	[(TiO_2_/Al_2_O_3_)^N^/Al_2_O_3_/(TiO_2_/Al_2_O_3_)^N^] (Cylindrical defect-mode cavity)	0.0065	N.A	N.A	0–300 °C
[64]	air/(Si/PS)^N^/SiO_2_/(Si/PS)^N^/glass substrate (Conventional Bragg-type defect cavity)	0.085	2216.6	0.0243	25–900 °C
[65]	alternating layers of Gallium Nitride (GaN) and Silicon Nitride (Si_3_N_4_) with double defect layers of air (Conventional double-defect cavity)	Mode1: 0.008 Mode2: 0.014	Mode1: 158.5 Mode2: 125.8	Mode1: 0.00127 Mode2: 0.00129	1000–1700 °C
Present work	[(Si/Gl)^6^(Gl/Si)^6^/Gl/(Si/Gl)^6^(Gl/Si)^6^] (Topological-mirror-supported Fabry–Pérot-type defect cavity)	−0.1177	~1.77 × 10^8^	1.52 × 10^4^	20–55 °C

**Table 4:** Correlated variations in the periodic-layer thicknesses at T = 35 °C.

Deviation (%)	*t_1Si_* (nm)	*t_1Gl_* (nm)	*t_2Si_* (nm)	*t_2Gl_* (nm)	*D_Gl_* (nm)	*λ_res_* (nm)	*T_peak_*	*FWHM* (nm)	*Q*
−3%	291	194	94.09	223.1	500	1356.99	0.749	~8.09 × 10^−6^	~1.68 × 10^8^
−1%	297	198	96.03	227.7	500	1374.02	0.992	~8.19 × 10^−6^	~1.68 × 10^8^
0%	300	200	97	230	500	1380.70	0.998	~7.75 × 10^−6^	~1.77 × 10^8^
+1%	303	202	97.97	232.3	500	1390.86	0.9811	~8.75 × 10^−6^	~1.59 × 10^8^
+3%	309	206	99.91	236.9	500	1407.59	0.148	~9.88 × 10^−6^	~1.42 × 10^8^

**Table 5:** Independent variations in the central glycerin-defect thickness at T = 35 °C.

Deviation (%)	*t_1Si_* (nm)	*t_1Gl_* (nm)	*t_2Si_* (nm)	*t_2Gl_* (nm)	*D_Gl_* (nm)	*λ_res_* (nm)	*T_peak_*	*FWHM* (nm)	*Q*
−3%	300	200	97	230	485	1367.35	0.1515	~1.01 × 10^−5^	~1.36 × 10^8^
−1%	300	200	97	230	495	1377.45	0.6889	~8.83 × 10^−6^	~1.56 × 10^8^
0%	300	200	97	230	500	1380.70	0.998	~7.75 × 10^−6^	~1.77 × 10^8^
+1%	300	200	97	230	505	1387.44	0.9067	~8.20 × 10^−6^	~1.69 × 10^8^
+3%	300	200	97	230	515	1397.31	0.8712	~8.05 × 10^−6^	~1.73 × 10^8^

## Data Availability

The raw data supporting the conclusions of this article will be made available by the authors on request.
